# Artificial intelligence-intracardiac echocardiography-guided pulsed field ablation of atrial fibrillation with variable-loop circular catheter

**DOI:** 10.1093/europace/euag212

**Published:** 2026-08-26

**Authors:** Fengwei Zou, Sarah Xu, Justin Song, Ariel Gidon, Aung Lin, Nikil Prasad, Nils Guttenplan, Nicola Tarantino, Ronghua Yang, Dhanunjaya Lakkireddy, Vincenzo Mirco La Fazia, Sanghamitra Mohanty, Andrea Natale, Vito Grupposo, Xiaodong Zhang, Luigi Di Biase

**Affiliations:** Montefiore-Einstein Center for Heart and Vascular Care, Montefiore Medical Center, Montefiore Health System, Albert Einstein College of Medicine, 111 E 210th street, Bronx, NY 10467, USA; Montefiore-Einstein Center for Heart and Vascular Care, Montefiore Medical Center, Montefiore Health System, Albert Einstein College of Medicine, 111 E 210th street, Bronx, NY 10467, USA; Montefiore-Einstein Center for Heart and Vascular Care, Montefiore Medical Center, Montefiore Health System, Albert Einstein College of Medicine, 111 E 210th street, Bronx, NY 10467, USA; Montefiore-Einstein Center for Heart and Vascular Care, Montefiore Medical Center, Montefiore Health System, Albert Einstein College of Medicine, 111 E 210th street, Bronx, NY 10467, USA; Montefiore-Einstein Center for Heart and Vascular Care, Montefiore Medical Center, Montefiore Health System, Albert Einstein College of Medicine, 111 E 210th street, Bronx, NY 10467, USA; Montefiore-Einstein Center for Heart and Vascular Care, Montefiore Medical Center, Montefiore Health System, Albert Einstein College of Medicine, 111 E 210th street, Bronx, NY 10467, USA; Montefiore-Einstein Center for Heart and Vascular Care, Montefiore Medical Center, Montefiore Health System, Albert Einstein College of Medicine, 111 E 210th street, Bronx, NY 10467, USA; Montefiore-Einstein Center for Heart and Vascular Care, Montefiore Medical Center, Montefiore Health System, Albert Einstein College of Medicine, 111 E 210th street, Bronx, NY 10467, USA; Montefiore-Einstein Center for Heart and Vascular Care, Montefiore Medical Center, Montefiore Health System, Albert Einstein College of Medicine, 111 E 210th street, Bronx, NY 10467, USA; Kansas City Heart Rhythm Institute, University of Missouri-Columbia, HCA Midwest Health, Overland Park, KS, USA; Texas Cardiac Arrhythmia Institute, St David’s Medical Center, Austin, TX, USA; Texas Cardiac Arrhythmia Institute, St David’s Medical Center, Austin, TX, USA; Texas Cardiac Arrhythmia Institute, St David’s Medical Center, Austin, TX, USA; Johnson and Johnson MedTech Electrophysiology, Johnson and Johnson, Irvine, CA, USA; Montefiore-Einstein Center for Heart and Vascular Care, Montefiore Medical Center, Montefiore Health System, Albert Einstein College of Medicine, 111 E 210th street, Bronx, NY 10467, USA; Montefiore-Einstein Center for Heart and Vascular Care, Montefiore Medical Center, Montefiore Health System, Albert Einstein College of Medicine, 111 E 210th street, Bronx, NY 10467, USA

**Keywords:** Atrial fibrillation, Pulsed field ablation, Variable-loop circular catheter, VARIPULSE, Electroanatomical mapping, Intracardiac echocardiography, CARTOSOUND FAM, Artificial intelligence

## Abstract

**Aims:**

Intracardiac echocardiography (ICE) facilitates left atrial (LA) reconstruction during atrial fibrillation (AF) ablation. The artificial intelligence–based CARTOSOUND FAM (AIFAM) module enables automated three-dimensional LA reconstruction without the need for a dedicated pre-ablation mapping catheter. While this workflow has been described previously in radiofrequency ablation, its application and outcome in pulsed field ablation (PFA) remain limited. The objective of this study is to evaluate the feasibility, safety, and arrhythmia-free outcomes of a no-pre-mapping AIFAM-guided workflow during PFA using the variable-loop circular catheter (VLCC).

**Methods and results:**

Patients undergoing PFA AF ablation using VLCC were screened and those using AIFAM-guided ablation without pre-map were included in the analysis. The LA shell was created using the AI-based ICE module from the right atrium without the need for a LA mapping catheter and was used as the only reference shell for PFA energy delivery. Baseline demographics, AF subtype, comorbidities, procedural details, and follow-up outcomes were analysed. Recurrence was assessed using a 60-day blanking period and Kaplan–Meier survival analysis. A total of 412 patients underwent AF ablation using VLCC during the study period. Among them, 210 patients (mean age 68.1 ± 10.2 years; 54.8% male) underwent AIFAM-guided ablation without pre-map and were analysed. Atrial fibrillation subtype was paroxysmal in 52% and non-paroxysmal in 48%. Concomitant AF ablation and left atrial appendage occlusion (LAAO) were performed in 76 patients (36%). The mean CHA_2_DS_2_-VASc score was 3.4 ± 1.7, and the mean left ventricular ejection fraction was 56.2 ± 11.1%. All patients underwent pulmonary vein isolation, with additional PF lesions including posterior wall isolation in 87.5% and superior vena cava ablation in 60.5%. Skin-to-skin procedure time and LA dwell time were 78.8 ± 29.1 min and 47.1 ± 18.8 min for ablation alone and 87.3 ± 22.9 min and 54.6 ± 13.0 min for concomitant AF ablation and LAAO. Acute procedural success was achieved in 100% of cases. One acute non-catheter-related vascular complication (0.5%), and no late complications were documented. Among 184 patients with at least one follow-up, freedom from arrhythmia survival after the 60-day blanking was 77.7% in paroxysmal AF and 71.8% in non-paroxysmal AF at 270 days.

**Conclusion:**

AIFAM enables safe and reliable LA reconstruction for successful PFA ablation, including concomitant AF ablation and LAAO using VLCC, and reduces the need for pre-ablation mapping in select patients undergoing index ablation. This strategy demonstrated high acute success, low complication rates, and favourable arrhythmia freedom in a contemporary cohort.

## Graphical Abstract

**Graphical Abstract:**
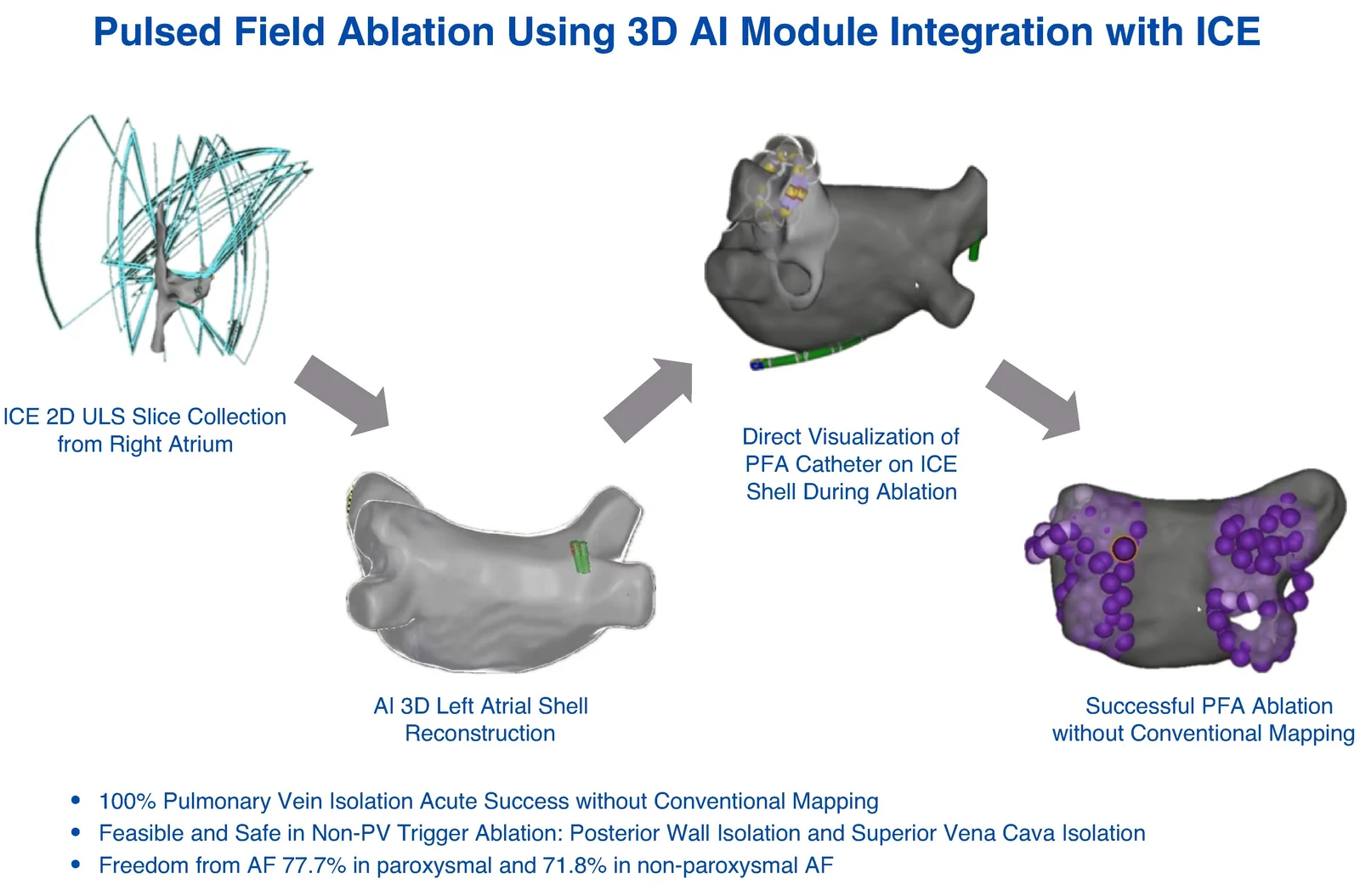

What's new?

- AIFAM guided PF ablation without pre-map using the VLCC was feasible and safe, with low acute complication rate and no late complications.
- AIFAM guided PF ablation using VLCC is effective and excellent in achieving a cute procedural success for PVI as well as non-PV targets including the PW and SVC.

## Introduction

The widespread adoption of intracardiac echocardiography (ICE) has significantly advanced cardiac imaging in modern atrial fibrillation (AF) catheter ablation.^1^ Intracardiac echocardiography enables real-time visualization of cardiac structures during transseptal puncture and catheter manipulation, facilitates early recognition of complications, and reduces fluoroscopy exposure. The CARTO™ 3 System (Biosense Webster, part of Johnson & Johnson MedTech, Irvine, CA, USA) is a three-dimensional electroanatomical mapping (EAM) platform that generates high-resolution 3D maps using magnetic localization technology. The CARTOSOUND™ FAM module integrates a deep learning–based artificial intelligence (AIFAM) algorithm that automates left atrial (LA) shell reconstruction using ICE without the need for a dedicated mapping catheter.^2^

This AIFAM module has demonstrated high fidelity in identifying key LA anatomical landmarks, including the pulmonary veins (PVs) and left atrial appendage (LAA), with measurements comparable to pre-procedural computed tomography and strong inter-operator reproducibility.^2^ In our prior experience incorporating AIFAM into radiofrequency (RF) ablation workflows, the elimination of conventional pre-ablation mapping with a dedicated mapping catheter was associated with reductions in overall procedure time, LA dwell time, and catheter exchange while maintaining high acute success and safety.^3^

Pulsed field ablation (PFA) has quickly emerged as a predominant energy choice for AF ablation due to its tissue-selective, favourable safety profile and efficient lesion formation properties.^4,5^ The VARIPULSE™ PFA system (Biosense Webster, part of Johnson & Johnson MedTech, Irvine, CA, USA) is a variable-loop circular catheter (VLCC) platform that enables safe and effective AF ablation and is fully integrated with the CARTO™ 3 and CARTOSOUND FAM systems. However, the feasibility and efficacy of AI-guided ICE-based no-pre-map PFA workflow have not been well described. In a recent European Heart Rhythm Association (EHRA) survey, wide heterogeneity exists in the methods applied for PFA in different centres, with ICE reported as a standard tool in only 13%.^6^ In this study, we aimed to evaluate the feasibility, safety profile, and arrhythmia-free outcomes of the AIFAM-guided, no-pre-map workflow during VLCC PFA ablation.

## Methods

### Study population

Patients undergoing pulsed field AF ablation using VLCC at a high-volume US centre were screened for this study. Among these, consecutive patients treated using the AIFAM no-pre-mapping workflow (described below) were included in the present analysis. Patients in whom conventional EAM with a dedicated mapping catheter was performed prior to ablation were not included.

### AIFAM-guided pulsed field ablation using variable-loop circular catheter

**Figure 1:**
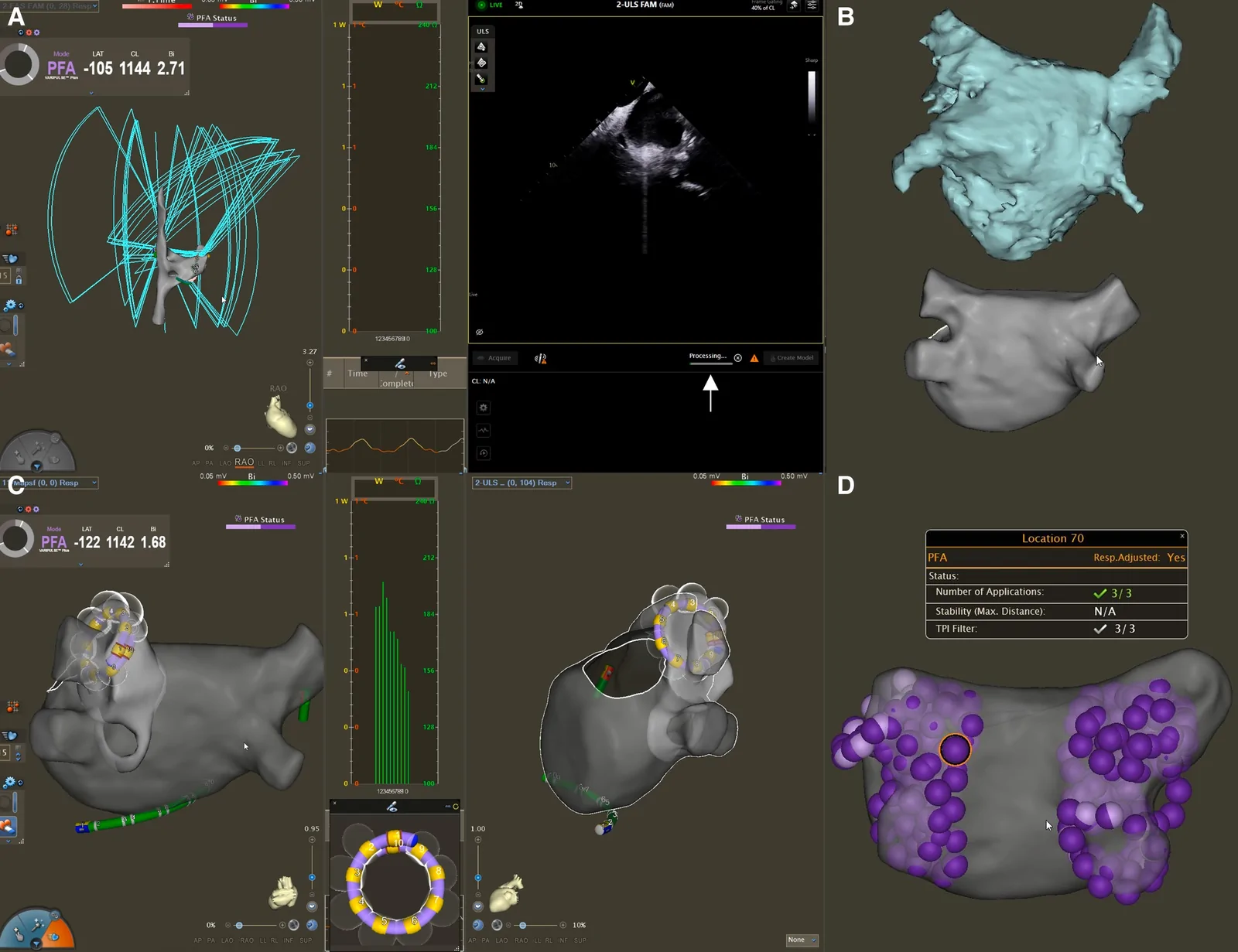
AIFAM ICE-guided ablation workflow with VLCC PFA system. AIFAM ICE-guided sample workflow. (*A*) ICE FAM slices were generated by rotating the ICE catheter in the right atrium. The left atrial 3D anatomical shell in this case was being generated by AI automatically (arrow showing processing). The automation process was 21 s for this case. For a better real-time example, see Supplementary material online, *Video S1*. (*B*) Direct comparison of the LA shell from cardiac CT (upper) and from AIFAM generation (lower). (*C*) VLCC position in the left superior pulmonary vein projected on the AIFAM-generated shell in standard posterior–anterior and left-lateral views on CARTO. Note that all electrodes of the VLCC had positive tissue-proximity indicators (triangle showing the TPI cloud). (*D*) Final ablation lesion set projected on the AIFAM-generated shell. A lesion tag was selected, showing successful delivery of all three PFA pulses and positive TPI contact in all pulses. Abbreviations: AI, artificial intelligence; CARTO, electroanatomic mapping system; CT, computed tomography; FAM, fast anatomical mapping; ICE, intracardiac echocardiography; LA, left atrium; PFA, pulsed field ablation; TPI, tissue proximity indicator; VLCC, variable-loop circular catheter.

All patients proceeded with AF ablation or concomitant AF ablation and left atrial appendage occlusion (LAAO) under uninterrupted anticoagulation without changes to routine institutional procedure workflow. All patients received general anaesthesia for the procedure. Vascular access was obtained under ultrasound guidance in the femoral veins, and a decapolar diagnostic catheter was positioned in the coronary sinus. Subsequently, an ICE catheter (SOUNDSTAR^TM^ or SOUNDSTAR CRYSTAL^TM^) was advanced into the right atrium (RA) and used to visualize the RA, tricuspid valve, fossa ovalis, coronary sinus, overall LA body, aortic valve, LAA, left superior PV, left inferior PV, right superior PV, and right inferior PV. The AIFAM module was then used to automatically reconstruct the LA anatomy without a mapping catheter before the initial PF energy delivery. Descriptions of the AIFAM algorithm as well as best practice recommended workflow to accurately and efficiently create the LA anatomy were also previously summarized.^7^ A graphic and video illustration of the AIFAM module integration with the VLCC is shown in *Figure 1*.

Transseptal puncture was performed under ICE and/or fluoroscopic guidance using standard techniques. Access to the left atrium was obtained using either a BRK™ transseptal needle (Abbott Laboratories, Abbott Park, IL, USA) in conjunction with a SafeSept® guidewire (Pressure Products Medical Supplies Ltd., San Pedro, CA, USA), a VersaCross® RF wire, or an NRG® RF transseptal needle (Baylis Medical, Marlborough, MA, USA). Transseptal access was performed through a deflectable sheath, including the VersaCross® sheath (Baylis Medical, Marlborough, MA, USA), VIZIGO™ (Biosense Webster, part of Johnson & Johnson MedTech, Irvine, CA, USA), or AGILIS™ (Abbott Medical, Abbott Park, IL, USA), at the operator’s discretion. The sheath was continuously flushed with heparinized saline throughout the procedure. After an initial intravenous bolus of unfractionated heparin, activated clotting time was maintained at ≥350 s with additional boluses during the entire LA dwell time. The VARIPULSE™ PFA catheter (Biosense Webster, part of Johnson & Johnson MedTech, Irvine, CA, USA) was then advanced into the LA via the deflectable sheath, and a tissue proximity indicator (TPI) was used to assess real-time catheter–tissue contact. For pulmonary vein isolation (PVI), at least four ablations were performed, with two at the ostium and two at the antrum of each PV. The catheter was slightly rotated relative to the initial position to cover any gaps between electrodes 1 and 10 between each ostial or antral lesion. Additional PF ablations at the roof, floor, ridge, or carina of the PVs were performed at the operator’s discretion. Further ablation of non-PV sites, including the posterior wall, superior vena cava (SVC), cavotricuspid isthmus (CTI), mitral isthmus, and anterior wall, using either VLCC or RF energy was selected based on the operator’s judgement. Catheter–tissue contact is assessed using ICE and TPI at all times, aiming to maximize TPI visualization for all electrodes whenever possible before delivery of each lesion. For SVC ablations, the VARIPULSE™ catheter was retracted into the RA within the sheath and readvanced to the SVC to deliver two lesions at the level of the pulmonary artery under direct ICE visualization. Acute success is defined as the entrance and exit block of each PV for PVI, the absence of any electrical potentials in the ablation of non-PV triggers at the end of ablation, and the confirmation of linear blocks using differential pacing.

### Concomitant atrial fibrillation ablation and left atrial appendage occlusion

For concomitant AF ablation and LAAO, either transesophageal echocardiography (TEE) or ICE was used to guide LAA anatomical assessment, LAAO device selection, and real-time monitoring during device deployment.^8^ In general, after left-side ablation was done, the deflectable ablation sheath was exchanged for the TruSteer™ steerable sheath (Boston Scientific, Marlborough, MA, USA) over a wire, and LAAO is performed using WATCHMAN FLX™ PRO devices. For SVC ablation after LAAO, the VARIPULSE™ catheter was retracted into the RA within the TruSteer sheath and readvanced to the SVC to deliver two lesions as described above. At the end of the procedure, protamine was given, sheaths and catheters were removed, and haemostasis was achieved.

### Follow-Up

Baseline demographic information, comorbidities, medication use, and procedure-related information, including skin-to-skin procedure time, fluoroscopy time, LA dwell time, AF ablation lesion sets, number of PF lesions, complications, and acute success, were summarized at the time of the procedure as part of an institutional registry. All patients received routine follow-up at 1 month or 45 days (TEE for concomitant AF ablation and LAAO to assess LAAO leaks), 6 months, 12 months, and yearly after the procedure. Continuous rhythm monitors were prescribed at the provider’s discretion and were reviewed at the following visit. Electrocardiograms (ECGs) were performed in all clinic visits, and patients were encouraged to contact the providers should symptoms occur to expedite rhythm confirmation.

Freedom from AF recurrence (AF, atrial tachycardia, or atrial flutter exceeding 30 s) was evaluated with or without the use of antiarrhythmic medications (AADs). Atrial arrhythmia recurrence occurring in the first 60 days after the ablation was considered to be within the blanking period. This study is approved by the institutional review board.

### Statistical analysis

Continuous variables were calculated as mean ± standard deviation and compared using independent *t*-tests. Categorical variables were presented as frequency (%) of the total cohort and were compared using χ^2^ tests. Freedom from atrial arrhythmia recurrence over time was analysed using Kaplan–Meier survival curves and utilizing the log-rank test. A two-sided *P* value of < 0.05 was considered statistically significant.

## Results

### Patient population

A total of 412 patients underwent AF ablation using VLCC during the study period. Among these, 210 consecutive patients underwent PFA using the AIFAM no-pre-map workflow and comprised the study cohort. Baseline demographic characteristics and comorbidities are summarized in *Table 1*. The mean age was 68.1 ± 10.2 years, and 45.2% were female. The mean body mass index was 30.6 ± 7.0 kg/m^2^. The mean CHA_2_DS_2_-VASc score was 3.4 ± 1.7. Common comorbidities in descending order of prevalence included hypertension (85.2%), hyperlipidaemia (75.2%), Type 2 diabetes mellitus (36.2%), congestive heart failure (34.8%), coronary artery disease (30.0%), chronic kidney disease/end-stage renal disease (20.0%), obstructive sleep apnoea (16.2%), and prior cerebrovascular accident (11.9%). Oral anticoagulation was prescribed in 98.6% of patients, with apixaban being the most common (89.5%). Patients not on pre-procedural anticoagulation received LAAO as a prior separate procedure. The LA diameter averaged 4.2 ± 0.7 cm, and left atrial volume index was 36.8 ± 14.2 mL/m^2^. The left ventricular ejection fraction was 56.2 ± 11.1%. Paroxysmal AF was present in 51.9% of patients.

**Table 1:** Baseline characteristics (*n* = 210)

Age	68.1 ± 10.2
Female	95 (45.2%)
BMI	30.6 ± 7.0
LVEF	56.2 ± 11.1
Left atrial diameter (cm)	4.2 ± 0.7
Left atrial volume (cm^3^)	72.2 ± 28.0
LAVi (cm^3^/m^2^)	36.8 ± 14.2
CHA_2_DS_2_-VASc score	3.3 ± 1.7
CHA_2_DS_2_-VA score	2.9 ± 1.6
CAD	63 (30.0%)
DM	76 (36.2%)
HLD	158 (75.2%)
HTN	179 (85.2%)
Obesity	98 (46.7%)
CHF	73 (34.8%)
CKD/ESRD	42 (20.0%)
OSA	34 (16.2%)
Chronic obstructive pulmonary disease	18 (8.6%)
CVA	25 (11.9%)
OAC	207 (98.6%)
Apixaban	188 (89.5%)
Rivaroxaban	16 (7.6%)
Dabigatran	1 (0.5%)
Warfarin	2 (1.0%)
AF type	
Paroxysmal	109 (51.9%)
Non-paroxysmal	101 (48.1%)

Values are presented as mean ± standard deviation or *n* (%). CHA_2_DS_2_-VASc and CHA_2_DS_2_-VA scores were calculated for baseline thromboembolic risk stratification. Non-paroxysmal AF includes persistent and long-standing persistent AF.

Abbreviations: AF, atrial fibrillation; BMI, body mass index; CAD, coronary artery disease; CHA_2_DS_2_-VASc, congestive heart failure, hypertension, age ≥ 75 years, diabetes mellitus, prior stroke/transient ischaemic attack/thromboembolism, vascular disease, age 65–74 years, and sex category; CHF, congestive heart failure; CKD, chronic kidney disease; CVA, cerebrovascular accident; DM, diabetes mellitus; ESRD, end-stage renal disease; HLD, hyperlipidaemia; HTN, hypertension; LAVi, left atrial volume index; LVEF, left ventricular ejection fraction; OAC, oral anticoagulation; OSA, obstructive sleep apnoea.

### Procedural characteristics

Procedural characteristics are summarized in *Table 2*. Atrial fibrillation ablation alone was performed in 134 patients (63.8%), and concomitant AF ablation and LAAO were performed in 76 patients (36.2%). Pulmonary vein isolation was performed and achieved in all patients (100%). Additional PF lesion sets included posterior wall isolation (PWI) in 87.6% and SVC ablation in 60.5%. Radiofrequency energy was generally used to perform linear ablations, including CTI ablation in 15 patients (7.1%) and mitral isthmus line ablation in two patients (1.0%).

**Table 2:** Procedure characteristics

Procedure duration (min)	81.9 ± 27.3
AF ablation	78.8 ± 29.1
Concomitant AF ablation + LAAO	87.3 ± 22.9
Fluoroscopy duration (min)	14.2 ± 7.7
AF ablation	12.7 ± 7.6
Concomitant AF ablation + LAAO	16.4 ± 7.2
LA dwell time (min)	49.8 ± 17.3
AF ablation	47.1 ± 18.8
Concomitant AF ablation + LAAO	54.6 ± 13.0
Number of complete PFA applications	25.6 ± 6.3
Number of incomplete PFA applications	1.1 ± 3.0
Lesion set	
PVI	210 (100.0%)
PWI	184 (87.6%)
SVC	127 (60.5%)
CTI (RF)	15 (7.1%)
Mitral isthmus line (RF)	2 (1.0%)

Values are presented as mean ± standard deviation or *n* (%). Procedural duration, fluoroscopy duration, and left atrial dwell time are reported for the overall cohort and separately for AF ablation-only procedures and concomitant AF ablation with left atrial appendage occlusion. Lesion sets refer to ablation lesion sets performed during the procedure. Cavotricuspid isthmus and mitral isthmus ablation were performed using radiofrequency ablation.

Abbreviations: AF, atrial fibrillation; CTI, cavotricuspid isthmus; LA, left atrium; LAAO, left atrial appendage occlusion; PFA, pulsed field ablation; PVI, pulmonary vein isolation; PWI, posterior wall isolation; RF, radiofrequency; SVC, superior vena cava.

The mean total skin-to-skin procedure duration for the overall cohort was 81.9 ± 27.3 min (median 74.0 min, interquartile range [IQR] 63.0–93.8). When stratified by procedural type, AF ablation-alone cases had shorter procedure times compared with concomitant AF ablation and LAAO (78.8 ± 29.1 vs. 87.3 ± 22.9 min, *P* = 0.021). The mean fluoroscopy duration for the overall cohort was 14.2 ± 7.7 min. Fluoroscopy time was shorter in AF-alone cases compared with concomitant AF ablation and LAAO (12.7 ± 7.6 vs. 16.4 ± 7.2 min, *P* = 0.001). A total of 26 AF ablations were performed without fluoroscopy. The mean LA dwell time was 49.8 ± 17.3 min overall. Similarly, AF-alone procedures had shorter LA dwell times compared with concomitant AF ablation and LAAO (47.1 ± 18.8 vs. 54.6 ± 13.0 min, *P* = 0.001). The mean number of complete PFA applications was 25.6 ± 6.3, with 1.12 ± 2.99 incomplete applications. Acute procedural success was achieved in all patients. One non-catheter-related vascular complication (0.5%) occurred (groin haematoma requiring manual compression the following day). No blood transfusion, surgical, or vascular intervention was required.

### Clinical outcomes

**Figure 2:**
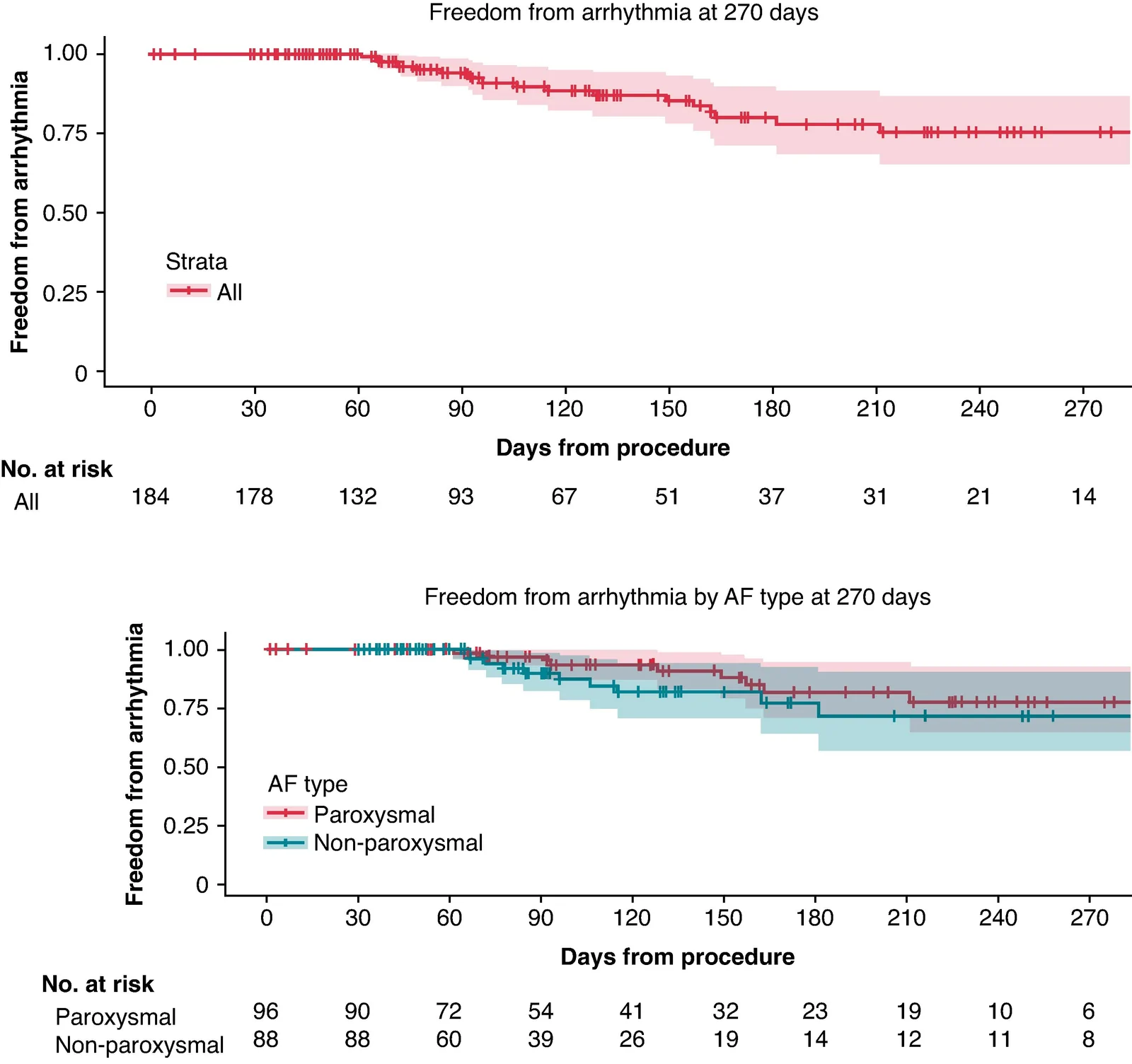
Freedom from atrial arrhythmia at 270 days. Kaplan–Meier estimates of freedom from atrial arrhythmia following ablation in the overall cohort (top) and stratified by AF type (bottom). At 270 days, freedom from atrial arrhythmia was 75.2% for the overall cohort, 77.7% in patients with paroxysmal AF and 71.8% in those with non-paroxysmal AF. Numbers at risk are shown below each plot.

Among 184 patients with at least one follow-up (mean follow-up duration 127.1 days), recurrence analysis was performed using a 60-day blanking period. Kaplan–Meier freedom from arrhythmia was 82.0% at 180 days and 75.2% at 270 days for the overall cohort, with 18.6% and 18.8% of patients remaining on AADs, respectively (*Figure 2A*). At 270 days, freedom from arrhythmia was 77.7% in patients with paroxysmal AF and 71.8% in those with non-paroxysmal AF (*Figure 2B*). No late complications were observed during follow-up.

## Discussion

The main findings of this study were as follows: (i) AIFAM-guided PF ablation without pre-map using the VLCC was feasible and safe, with a low acute complication rate and no late complications; (ii) AIFAM-guided PF ablation using VLCC is effective and excellent in achieving acute procedural success for PVI as well as non-PV targets, including the PW and SVC; and (iii) freedom from atrial arrhythmia was promising in both paroxysmal and non-paroxysmal AF, reaching 77.7% and 71.8% at 270 days, respectively. To the best of our knowledge, this study represents the first study and one of the largest real-world cohorts that evaluated both acute safety and success and freedom from atrial arrhythmia at 270-day follow-up of the AIFAM-guided PF ablation workflow without pre-ablation mapping using the VLCC PFA platform.

Catheter ablation has progressively become a first-line rhythm control strategy for patients with AF, supported by the 2023 American College of Cardiology/American Heart Association/American College of Clinical Pharmacy/Heart Rhythm Society (ACC/AHA/ACCP/HRS) Guideline for the Diagnosis and Management of Atrial Fibrillation.^9^ The introduction of PFA has further accelerated adoption by offering tissue selectivity and a favourable safety profile compared with conventional thermal technologies. Among currently available systems, the mapping-capable VLCC represents a robust platform fully integrated within the CARTO EAM environment. The pivotal inspIRE and admIRE trials have demonstrated the safety and effectiveness of PVI using the VLCC PFA system in paroxysmal AF, reporting 12-month freedom from symptomatic atrial arrhythmia of 75.4–78.9%, with a mean total procedure time of ∼70–80 min.^10,11^ Subsequent observational reports have further supported procedural feasibility and acute safety with VLCC in real-world applications, especially after the adoption of a 30 mL/min irrigation flow during PF delivery.^12–15^ Currently, most existing data focused primarily on PVI in treating paroxysmal AF, and large prospective trials evaluating non-PV lesion sets, such as PWI or SVC ablation, remain limited. In our present study, the patient cohort included close to 50% non-paroxysmal AF, with a high prevalence of PWI (>80%) and SVC ablation (>60%) as non-PV targets, and 36.2% of cases were concomitant AF ablation and LAAO procedures.

### Feasibility and acute success of the AIFAM-guided no-pre-map workflow

A key feature of the present study is the elimination of pre-ablation contact mapping through integration of AI-assisted ICE-based anatomical reconstruction using the CARTOSOUND FAM module. Early validation studies assessed the geometric accuracy of AI-generated LA reconstructions by comparing PV ostial diameters, LAA dimensions, and carina-to-carina distances with pre-procedural cardiac computed tomography (CT), demonstrating strong concordance and low inter-operator variability.^16^ In direct comparisons between AIFAM-derived anatomy and conventional contact-based EAM during ablation, the median distance between the ablation catheter position and the AIFAM shell was <5 mm across all PVs. Furthermore, key anatomical landmarks, including the fossa ovalis used for transseptal access, were accurately reproduced, supporting reliable guidance during critical procedural steps.^16^ Our previous study evaluating acute and 1-year outcomes of AIFAM integration during RF ablation showed procedural efficiency improvements by reducing total procedure time and LA dwell time without compromising long-term rhythm control efficacy.^3^

In the PFA era, a recent Italian multicentre experience also demonstrated that the AIFAM ICE reconstruction is feasible and associated with reductions in mapping time and LA dwell time compared with conventional EAM.^15^ Although a smaller cohort of only 64 patients underwent AIFAM-guided no-pre-map ablation with VLCC and fewer non-PV triggers were targeted, 100% acute success was achieved, similar to our report here.^15^ In addition to demonstrating feasibility and excellent acute success, our workflow maintained favourable efficiency despite frequent utilization of non-PV lesion sets. The mean total procedure time of 81.9 min and mean LA dwell time of 49.8 min were achieved in a cohort in which the majority of patients underwent both PVI and PWI. Compared with previously reported procedural times in pivotal PFA trials that largely focused on PVI-only strategies, our procedural duration remains comparable despite a more extensive ablation approach.^10,11^ These findings suggest that the streamlined no-pre-map AIFAM workflow, combined with the integrated VLCC platform, enables efficient execution of both standard and additional lesion sets without a significant increase in LA dwell time and procedure time. Last but not the least, the VLCC platform enables simultaneous mapping and real-time matrix acquisition within the CARTO system as the catheter is manipulated within the LA, allowing continuous geometry refinement and contour optimization on top of the AIFAM-generated ICE anatomy. These features further enhance anatomical fidelity.

### Arrhythmia freedom using AIFAM-guided atrial fibrillation ablation with variable-loop circular catheter

To our knowledge, this is one of the first studies reporting 270-day freedom from atrial arrhythmia in a real-world VLCC PFA cohort. Using a 60-day blanking period, freedom from arrhythmia reached 82.0% at 180 days and 75.2% at 270 days for the overall cohort (*Figure 2*), with 18% of patients remaining on AADs. These favourable outcomes were observed in both paroxysmal and non-paroxysmal AF and are on par with the 12-month outcomes reported in the insPIRE and admIRE trials for patients with paroxysmal AF.^10,11^ We previously demonstrated in an RF cohort that elimination of pre-map contact mapping using AIFAM preserved anatomical fidelity and did not compromise acute or long-term arrhythmia outcomes.^3^ The present study extends those findings into the PFA era. From a mechanistic standpoint, adequate tissue–catheter contact remains a critical determinant of durable lesion formation, even in the PFA era. In our workflow, real-time TPI assessment provided immediate feedback regarding catheter–tissue engagement. Together with the VLCC’s ability to continuously update the CARTOSOUND FAM shell as the catheter was manipulated, dynamic refinement of LA geometry at the ablation target is seen from our experience. This combination of real-time contact assessment and geometry optimization may enhance lesion consistency and contribute to durable isolation, especially during PWI, where surface contour variability is greater. Together, these integrated mapping and contact features likely support the favourable arrhythmia-free survival observed in this cohort despite elimination of pre-ablation contact mapping. We believe these data suggest that AI-based anatomical reconstruction maintains adequate spatial accuracy to support durable lesion formation across energy modalities.

### Safety profile

Safety remains critical even in the PFA era. Early registry data from REAL-AF and VARIPURE demonstrated low acute complication rates with VLCC use,^12,17^ consistent with our findings. In our cohort, no acute or late major complications were observed, including no atrio-oesophageal fistula despite a high prevalence of PWI. Although PFA is mechanistically nonthermal, one recent isolated post-marketing report in the MAUDE database has described a case of atrio-oesophageal fistula temporally associated with the Affera PFA catheter.^18^ While causality cannot be definitively established and attributed to PF energy, the reporting underscores the importance of continued vigilance as PFA adoption expands. Furthermore, a recent dual-energy PFA study has demonstrated transient luminal oesophageal temperature rises during PWI, even when PFA is delivered in a nominally nonthermal mode.^19^ Although no transmural injury or fistula formation was identified in subsequent endoscopy, these findings suggest that oesophageal temperature elevations may occur under certain conditions. In our cohort, despite frequent PWI, no oesophageal injury or fistula was detected. Nevertheless, given the emerging data, careful attention to PW energy delivery and ongoing post-market surveillance remains essential.

In our study, no cerebrovascular accident (CVA) was observed during the follow-up period. This is particularly relevant given prior concerns regarding thromboembolic risk and microembolic signals reported with certain VLCC PFA platforms.^20,21^ In our cohort, the majority of cases utilized the updated 30 mL/min irrigation strategy, which has largely replaced the earlier 4 mL/min irrigation approach. While the present study is not powered to draw definitive mechanistic conclusions, the absence of clinical cerebrovascular events in a cohort with a higher mean number of PF applications (25.6 ± 6.3) is reassuring. In the context of large-scale post-market registries, our findings are also consistent with the growing body of evidence supporting the safety of VLCC PFA. The VARIPURE and REAL-AF registries have similarly demonstrated low rates of tamponade, stroke, and vascular complications.^12,13^ Together, these evidence support that a no-pre-map AI-assisted VLCC PFA workflow can be performed with a favourable safety profile, even in the setting of lesion delivery beyond PVI.

## Limitations

This study has several limitations. First, this was a single-centre observational experience without a direct randomized comparator group, as the purpose of this study is to mainly demonstrate the safety, feasibility, procedural workflow, and early rhythm outcomes of this AI-ICE integrated approach. It is also important to note that AI-ICE-based FAM can only assist with geometry construction and does not replace the functional information of substrate mapping, especially in redo ablations. Although the standardized workflow enhances internal consistency, it may limit generalizability. Second, follow-up was at 9-month assessment due to the novel nature of the VLCC platform, and longer-term durability beyond 1 year remains to be determined.

## Conclusions

AIFAM ICE-guided no-pre-map workflow with the VLCC PFA system achieved high procedural efficiency, safety, acute success, and promising arrhythmia-free survival at 9 months in a real-world AF population. These findings support the feasibility and effectiveness of eliminating pre-ablation contact mapping in the PFA era while underscoring the need for continued evaluation of long-term durability and safety.

## Supplementary Material

euag212_Supplementary_Data

## Data Availability

Data available on reasonable request.

## References

[1] Peichl P, Tzeis S, Skowronska M, Asvestas D, Baran J, Casella M et al Intracardiac echocardiography during invasive electrophysiological procedures. A scientific statement of the European Heart Rhythm Association of the ESC, and the European Association of Percutaneous Cardiovascular Interventions of the ESC. Europace 2026;28:euag059. 41973950 10.1093/europace/euag059 PMC13305637

[2] Di Biase L, Zou F, Lin A. Feasibility of three-dimensional artificial intelligence algorithm integration with intracardiac echocardiography for left atrial imaging during atrial fibrillation catheter ablation. Europace 2023;25:37477946. 10.1093/europace/euad211 PMC10403247 37477946

[3] Zou F, Xu S, Nagraj S, Mathai S, Gidon A, Kyaw NYW, et al Atrial fibrillation ablation using 3D artificial intelligence module integration with intracardiac echocardiography. Europace 2026;28:euag009. 41546622 10.1093/europace/euag009 PMC12950898

[4] Tzeis S, Gerstenfeld EP, Kalman J, Saad EB, Sepehri Shamloo A, Andrade JG et al 2024 European Heart Rhythm Association/Heart Rhythm Society/Asia Pacific Heart Rhythm Society/Latin American Heart Rhythm Society expert consensus statement on catheter and surgical ablation of atrial fibrillation. Europace 2024;26:euae043. 38587017 10.1093/europace/euae043 PMC11000153

[5] Chun KRJ, Miklavčič D, Vlachos K, Bordignon S, Scherr D, Jais P et al State-of-the-art pulsed field ablation for cardiac arrhythmias: ongoing evolution and future perspective. Europace 2024;26:euae134. 38848447 10.1093/europace/euae134 PMC11160504

[6] Bergonti M, Mills MT, Roten L, Ruwald MH, Metzner A, Conte G et al Pulsed field ablation for atrial fibrillation ablation: a European Heart Rhythm Association survey. Europace 2025;27:euaf294. 41363248 10.1093/europace/euaf294 PMC12686987

[7] Di Biase L, Grupposo V, Marazzato J, Tarantino N, Haiman G. LA workflow recommendations for intracardiac echocardiography integration with artificial intelligence 3D algorithm. Europace 2023;25:euad122.129. 10.1093/europace/euad211 PMC10403247 37477946

[8] Potpara T, Grygier M, Häusler KG, Nielsen-Kudsk JE, Berti S, Genovesi S et al Practical guide on left atrial appendage closure for the non-implanting physician: an international consensus paper. Europace 2024;26:euae035. 38291925 10.1093/europace/euae035 PMC11009149

[9] Joglar JA, Chung MK, Armbruster AL. ACC/AHA/ACCP/HRS guideline for the diagnosis and management of atrial fibrillation. J Am Coll Cardiol 2023;83:109–279. 38043043 10.1016/j.jacc.2023.08.017 PMC11104284

[10] Reddy VY, Calkins H, Mansour M, Wazni O, Di Biase L, Bahu M et al Pulsed field ablation to treat paroxysmal atrial fibrillation: safety and effectiveness in the AdmIRE pivotal trial. Circulation 2024;150:1174–86. 39258362 10.1161/CIRCULATIONAHA.124.070333 PMC11458102

[11] Duytschaever M, De Potter T, Grimaldi M, Anic A, Vijgen J, Neuzil P et al Paroxysmal atrial fibrillation ablation using a novel variable-loop biphasic pulsed field ablation catheter integrated with a 3-dimensional mapping system: 1-year outcomes of the multicenter inspIRE study. Circ Arrhythm Electrophysiol 2023;16:e011780. 36735937 10.1161/CIRCEP.122.011780 PMC10026968

[12] Porterfield C, Krishnan K, Saleem M. Low acute complication rates with a variable loop pulsed field ablation catheter: early real-world experience from the REAL-AF registry. Heart Rhythm Feb 2026;23:e295–7. 10.1016/j.hrthm.2025.10.027 41110532

[13] Sommer P, Kronborg MB, Sebag F. Workflow and sedation choice with the PFA variable-loop circular catheter in real-world AF procedures: insights from the prospective multi-centre VARIPURE clinical study. Europace 2026;28:euag025. 41655229 10.1093/europace/euag025 PMC12930087

[14] Andria N, Abuiznait Z, Saad M, Yousef S, Keselman S, Marai I. Single-center real world experience with the VARIPULSE platform for pulsed field ablation of atrial fibrillation, atrial flutter, and redo procedures. J Clin Med Dec 2025;15:28. 10.3390/jcm15010028 PMC12786650 41517277

[15] Dello Russo A, Valeri Y, Ciconte G, Schiavone M, Compagnucci P, Di Monaco A et al AI-assisted 3D intracardiac echocardiography for pulsed field ablation of atrial fibrillation using a novel variable loop circular catheter: a multicenter evaluation. J Clin Med 2025;14:7249. 41156118 10.3390/jcm14207249 PMC12565322

[16] Hanumanthu BK, Oranefo J, Patel N. Accuracy of deep learning based Cartosound fam of the left atrium compared to electroanatomical mapping. Heart Rhythm 2024;21:S624.

[17] Scherr D, Bessière F, Kronborg MB, Sebag F, Sohns C, Pappone C et al Effectiveness of AF pulsed field ablation with a variable loop circular catheter: 12-month VARIPURE results. Europace 2026;28:euag082. 41968105 10.1093/europace/euag082 PMC13303075

[18] FDA MAUDE Adverse Event Report. https://www.accessdata.fda.gov/scripts/cdrh/cfdocs/cfmaude/detail.cfm?mdrfoi__id=23733351&pc=QZI

[19] Fernandez-Ferro G, Cekaj E, Benz AP, Prengel B, Seidel P, Mollnau H et al Transient esophageal temperature heating observed during pulsed field ablation for atrial fibrillation. Heart Rhythm 2026;23:805–6. 41500485 10.1016/j.hrthm.2025.12.035

[20] Laskov V, Hozman M, Malikova H, Lauer D, Kremenova K, Herman D et al Cerebrovascular ischemic lesions after pulsed field ablation for atrial fibrillation using variable-loop ablation catheter. JACC Clin Electrophysiol 2026;12:814–23. 41575412 10.1016/j.jacep.2025.12.018

[21] Pagnoni M, Caranzano L, Teres C, Ascione C, Le Bloa M, Antiochos P et al Difference in cerebral microembolization with multielectrode pentaspline or Variable-loop circular PFA systems. J Cardiovasc Electrophysiol 2026;37:199–202. 41361678 10.1111/jce.70218 PMC12794735

